# Focal Cryoablation of Prostate: A Review

**DOI:** 10.1100/tsw.2008.56

**Published:** 2008-04-20

**Authors:** Duke K. Bahn, Paul D. Silverman

**Affiliations:** Prostate Institute of America, Community Memorial Hospital, Ventura, California, USA

**Keywords:** prostate cancer, focal therapy, cryoablation

## Abstract

Current treatment options for men with early localized prostate cancer are either some form of radical therapy or active surveillance. Radical therapy is usually associated with significant adverse effects that might jeopardize a man's quality of life. Some observers believe that PSA screening has resulted in the over diagnosis and over treatment of prostate cancer. Many men are being diagnosed with an early stage, small volume, unifocal or unilateral prostate cancer but are reluctant to accept watchful waiting or active surveillance. Focal cryoablation is the less than complete ablation of the gland with ice. Based on review of the limited amount of material available in the current literature, focal cryoablation can provide acceptable cancer control while preserving sexual potency and urinary continence. Focal cryoablation may fill a void in the therapeutic options available to patients with unifocal or unilateral prostate cancer who have a strong desire to maintain their quality of life.

## INTRODUCTION

The relatively slow-growing nature of clinically localized prostate cancer has refuted the current established treatment options for this disease. It is further complicated by stage migration and lead time bias associated with early-detected, small-volume cancers secondary to the popularity of Prostate Specific Antigen (PSA) screening. Many believe that PSA screening results in overdiagnosis and overtreatment of prostate cancer. This argument is supported by the fact that about one-third of men over the age of 50 will display incidental prostate cancer at autopsy, but only 10–16% will develop invasive prostate cancer during their lifetime, and only 2.5% will die from it[1,2,3].

Current treatment options for prostate cancer, other than active surveillance, are limited to entire prostate gland destruction through removal (radical prostatectomy), radiation (external beam, brachytherapy, or combination), or thermal ablation (cryoablation, high-intensity focused ultrasound, or radiofrequency).

Multiple large single institution case studies and prospective trials have reported acceptable treatment efficacy and morbidity of total cryoablation of the prostate[4,5,6,7]. The clinical outcomes after cryoablation measured as a biochemical disease-free status or negative biopsy rates are comparable to radical prostatectomy and radiation therapy in men with intermediate- and high-risk disease with significantly less morbidity[8].

Focal cryotherapy is defined as the less-than-complete ablation of the prostate gland with ice. A known tumor site (lobe) is aggressively treated, but the contralateral lobe of prostate tissue and surrounding structures are spared. This method offers targeted local cancer control, but preserves sexual potency and urinary continence in those whose cancer is believed to be unilateral in location.

In the PSA era, many cancers are detected at an early, organ-contained stage and could be confined to one lobe of the prostate. As many as 35% of clinically localized prostate cancers are unifocal and could be candidates for focal therapy[9]. A tumor less than 0.5 cc is used as a criterion for low-volume disease; this may not require any type of intervention. Others argue that even tumors smaller than 0.5 cc may be clinically aggressive and may require an appropriate intervention. It is indeed a burden to identify proper candidates for focal therapy.

## PATIENT SELECTION

Optimal patient selection criteria are not clearly identified and agreed in the urology field. However, it is essential that one must have unifocal or unilateral prostate cancer that is clearly identified and confirmed by gray scale and/or color Doppler transrectal ultrasound with targeted and sextant biopsy, in addition to initial extended or saturation biopsy. If an unexpected cancer was found in the other lobe by the repeated biopsy, regardless of the size of the lesion, the patient would not a candidate. Low-risk prostate cancers are preferred (PSA <10, Gleason <7, stage <T2b, number of positive cores <4, percentage of tumor invasion <50%), but moderate- to high-risk cancer in men with medical comorbidities could be considered. Only unilaterality, not preoperative PSA level or tumor differentiation, would be considered. Men with T3 stage (such as extracapsular extension or seminal vesicle invasion), as long as it is proven to be unilateral involvement, can be candidates after thorough counseling, since this may carry a risk of incomplete cryablation of the tumor that may end up with nothing but a tumor debulking process.

Candidates' prime concerns and priorities include maintaining quality of life and avoiding toxicity over cancer eradication. Candidates should have medical literacy that allows for informed consent regarding oncologic limitations of the procedure.

## METHODS

The cryoablation procedure is similar to that originally described by Onik et al.[10]. It consists of utilizing two freeze and thaw cycles, placing an FDA-approved urethral warming device, placing multiple cryoprobes percutaneously under ultrasound guidance, and using argon gas as a cryogenic material to create ice. However, by reducing the number of cryoprobes and adjusting their placement, it is possible to perform a focal ablation that destroys only a targeted portion of the prostate and periprostatic tissue adjacent to the treatment zone[11]. If a seminal vesicle invasion was proven, it would also be frozen by advancing one of the probes towards the distal segment of the lumen of the seminal vesicle. Usually two to four cryoprobes are used, depending on the size of the lesion and the size of the lobe. A single probe may be placed in the contralateral side near the urethra and external sphincter in case heating is necessary to protect these organs (simultaneous heating and cooling). This can be a useful technique if the prostate gland volume is small. The combination of aggressive freezing at targeted locations within the prostate while maintaining the integrity of the urethra, external sphincter, and contralateral lobe, including the neurovascular bundle, is the premise of focal cryoablation.

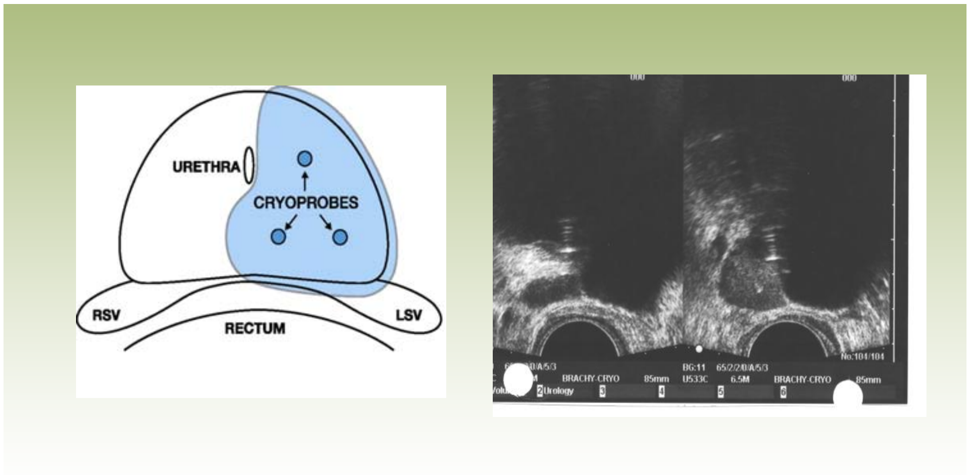

*Figure courtesy of the Prostate Institute of America.*

A PSA should be taken once every 3 months for 1 year and every 6 months thereafter. A biopsy is encouraged at 6 months, 1 year, 2 years, 5 years, and anytime there is a PSA elevating trend. Patients should fill out a questionnaire regarding sexual potency and urinary continence status at each visit without physician influence.

## RESULTS

To date, there are three peer-reviewed publications on this subject[12,13,14]. The number of patients studied is small, only 77 subjects combined. The mean follow-up is also short, in the range of 28–70 months

Various definitions of biochemical failure have been used to evaluate the clinical outcomes. These include the ASTRO (American Society for Therapeutic Radiology Oncology) definition that is three consecutive PSAs rising, the Phoenix definition that is the PSA nadir plus 2, and PSA nadir less than 50% of the preoperative level. The biochemical disease-free survival rates range from 84–95%.

A biopsy was performed when biochemical failure was suspected or as a protocol regardless of PSA levels. With every definition used, these three papers report fairly similar outcomes: 71 of the 77 men had undergone postcryotherapy biopsy with cancer identified in four patients (6%). All except one of these residual cancers were seen in the untreated lobe.

No major complications, such as rectal injury, urethral stricture, urethral sloughing, abscess, and peri- or postoperative complications were reported in any of these series. There was no reported case of urinary incontinence.

| | Onik (TCRT, August 2004) | Bahn (Endourology, Sep 2006) | Lambert (J Urol, June 2007) |
|---|---|---|---|
| Number | N = 21 | N = 31 | N = 25 |
| Follow-up | 24-105 months (mean = 50 m) | 8-107 months (mean = 70 months) | 9-72 months (mean = 28 months) |
| Stable PSA | 95% | 26/28 = 92.9% | 21/25 = 84% |
| Biopsy:Negative | N = 19; all negative | 26/27: 96.3% | 22/25: 88% |
| Potency Preservation | 17/21 = 80% | None: 3/27 = 9%<br>Partial: 11/27 = 41%<br>Full: 13/27 = 48%<br>(Partial + Full: 89%) | 17/24 = 71% |
| Age at procedure;<br>Age at follow-up | N/A<br>N/A | 63 years (procedure)<br>69 years (follow-up) | 68 years |
| Other Complications (incontinence, fistula) | None | None | None |

*Figure courtesy of the Prostate Institute of America.*

## DISCUSSION

Some observers believe that screening for PSA results in overdiagnosis and overtreatment of prostate cancer. The justification for screening and treatment is still accepted due to the fact that almost 30,000 men die from this disease every year in the U.S. Cryotherapy has evolved over the years as an accepted cancer treatment option since the published clinical outcome results are comparable to that of external beam radiation therapy and brachytherapy[7,8]. Unfortunately, in cryotherapy as in other conventional therapy, there are known potential side effects, mainly sexual dysfunction and urinary incontinence, which pose serious quality of life issues. In the PSA era, most prostate cancers are detected in the early stage, are frequently organ confined, and possibly localized in one lobe. Therefore, focal cryotherapy is attractive to many men who have a small and solitary cancer that may not require aggressive therapy.

Many patients who have small cancers are advised to forego aggressive treatment and instead undergo active surveillance management. This method is justified by the fact that prostate cancer grows slowly. However, there is always a possibility that this may result in missing a window of opportunity for cure. Focal cryotherapy may be a good compromise for men who are not comfortable with active surveillance, but who are also not comfortable with any type of radical treatment that may risk their quality of life.

It is the burden of the practitioner to identify the optimal candidate. Even though there are well-accepted criteria of low-risk disease, a confirmation of truly unilateral or unifocal disease is far from perfect. Multiple sets of biopsies and improved imaging studies, including color-Doppler transrectal ultrasound, may improve the cancer detection, but it is still possible that we may not detect a small biologically aggressive cancer that may be spared with this type of intervention. A patient must understand this risk that he is carrying.

There is limited information in literature on focal cryotherapy of clinically localized prostate cancer. To date, there are three peer-reviewed publications on this subject[12,13,14]. These are single institution experiences and there are no randomized clinical trial results. The number of patients studied is small, only 77 subjects combined. The mean follow-up is also short (28–70 months).

There is no consensus concerning the definition of biochemical disease-free survival after local therapy. The postradical prostatectomy, PSA definition of 0.2–0.4 ng/ml, would not be applicable to focal therapy. In radiotherapy, ASTRO has defined PSA progression as three consecutive PSAs rising or a PSA nadir over 1.0 mg/ml. Recently, the Phoenix definition has been introduced as a PSA nadir + 2. These definitions could be applied in focal therapy.

The PSA failure definitions used in these three papers varied. Most of them used the ASTRO definition and one paper analyzed its data using the Phoenix definition, and also PSA nadir 50% of the preoperative level. Regardless, the reported biochemical disease-free rates were encouraging, ranging from 84–95%.

Based on these series, the success of the procedure is probably best defined by follow-up biopsy; 71 patients have undergone one or more biopsies. Four positive biopsies were seen: one from the frozen lobe and three from the untreated lobes. All four patients received repeat cryoablation and currently have no evidence of disease. This finding may re-emphasize the criticism that prostate cancer is a multifocal disease; therefore, a focal treatment may not be logical. Most patients underwent two, rather extensive, preoperative biopsies to prove that their cancer was a unilateral disease. However, it is still possible that only the index tumor was detected and treated, and the other microscopic or small-volume tumors in the contralateral lobe were left untreated. Ohori et al.[15] presented data based on 1,000 radical surgery specimens, stating that the largest focus of cancer represented 80% of all the cancer volume. More than 90% of patients with extracapsular extension were from the largest focus. The study concluded that focal ablation of the index tumor will reduce 80% of tumor burden and extracapsular extension would be controlled in more than 90%[15].

The reported potency preservation, with or without pharmaceutical aid, was in the range of 71–89%. This is better than the rate reported in the bilateral nerve-sparing radical prostatectomy series, which reported 21–63%[16,17,18]. Focal cryotherapy is perhaps most compatible with unilateral nerve-sparing radical surgery, which is associated with potency preservation rates at 13–64%[19].

There are no published reports on quality of life following focal cryotherapy and only a few reports following total cryotherapy[20,21]. These data compared favorably with those of other treatment modalities for prostate cancer. At 1 year, most men return to presurgical functioning status in every area with the exception of sexual function. With focal cryotherapy, which is associated with a 71–89% sexual potency preservation rate, patients are expected to have improved rates of function return, and higher satisfaction.

## CONCLUSION

Focal cryosurgery represents a modification of the whole-gland approach and appears to offer acceptable oncologic efficacy with reduced treatment-related adverse events. Current data on focal cryotherapy are limited to determining the incidence or consequences of treatment failure. However, this procedure is minimally invasive and proven to be safe. The risk of incomplete eradication of cancer is likely to be small in the appropriately selected population of men with low-volume and low-risk prostate cancer. It is precisely this population of men who are presently confounded by the choice between active surveillance and more complex whole-gland treatment. Focal ablation is likely to be attractive to these men and, therefore, clinical research into improved prostatic imaging and large-scale, long-term, follow-up studies should be encouraged.
